# Ivermectin Treatment and Sanitation Effectively Reduce *Strongyloides stercoralis* Infection Risk in Rural Communities in Cambodia

**DOI:** 10.1371/journal.pntd.0004909

**Published:** 2016-08-22

**Authors:** Armelle Forrer, Virak Khieu, Christian Schindler, Fabian Schär, Hanspeter Marti, Meng Chuor Char, Sinuon Muth, Peter Odermatt

**Affiliations:** 1 Swiss Tropical and Public Health Institute, Basel, Switzerland; 2 University of Basel, Basel, Switzerland; 3 National Centre for Parasitology, Entomology and Malaria Control, Ministry of Health, Phnom Penh, Cambodia; Baylor College of Medicine, UNITED STATES

## Abstract

**Background:**

*Strongyloides stercoralis* is the only soil-transmitted helminth with the ability to replicate within its host, leading to long-lasting and potentially fatal infections. It is ubiquitous and its worldwide prevalence has recently been estimated to be at least half that of hookworm. Information on the epidemiology of *S*. *stercoralis* remains scarce and modalities for its large-scale control are yet to be determined.

**Methodology/Principal Findings:**

A community-based two-year cohort study was conducted among the general population in a rural province in North Cambodia. At each survey, participants infected with *S*. *stercoralis* were treated with a single oral dose of ivermectin (200μg/kg BW). Diagnosis was performed using a combination of the Baermann method and Koga agar plate culture on two stool samples. The cohort included participants from eight villages who were either positive or negative for *S*. *stercoralis* at baseline. Mixed logistic regression models were employed to assess risk factors for *S*. *stercoralis* infection at baseline and re-infection at follow-up. A total of 3,096 participants were examined at baseline, revealing a *S*. *stercoralis* prevalence of 33.1%. Of these participants, 1,269 were followed-up over two years. Re-infection and infection rates among positive and negative participants at baseline were 14.4% and 9.6% at the first and 11.0% and 11.5% at the second follow-up, respectively. At follow-up, all age groups were at similar risk of acquiring an infection, while infection risk significantly decreased with increasing village sanitation coverage.

**Conclusions/Significance:**

Chemotherapy-based control of *S*. *stercoralis* is feasible and highly beneficial, particularly in combination with improved sanitation. The impact of community-based ivermectin treatment on *S*. *stercoralis* was high, with over 85% of villagers remaining negative one year after treatment. The integration of *S*. *stercoralis* into existing STH control programs should be considered without further delay.

## Author Summary

*Strongyloides stercoralis* is an intestinal parasite that can replicate within its host, leading to long-lasting and potentially life-threatening infections. Occurring worldwide, it thrives where the climate is warm and sanitation is poor. *S*. *stercoralis* is not well detected by standard field techniques and its global prevalence is largely underestimated. Strongyloidiasis is arguably the most neglected of neglected tropical diseases. No control strategy currently exists for it. Ongoing control programs against other similar parasites regularly deliver treatment that has little effect on *S*. *stercoralis*. This parasite is a major public health issue in Cambodia, where prevalence rates of up to 40% were recently found. We diagnosed, treated and followed-up (over two years) more than 1,200 villagers in rural Cambodia to assess the impact of ivermectin on *S*. *stercoralis* at community level and assess whether *S*. *stercoralis* control could be integrated into existing programs. Our results indicate that ivermectin treatment was highly beneficial, with more than 85% of participants testing negative one year after treatment. All ages were at similar risk of acquiring an infection. The effect of community-based treatment was enhanced by increasing village sanitation coverage. We conclude that *S*. *stercoralis* control is feasible and strategies should be designed without further delay.

## Introduction

Infection with *Strongyloides stercoralis*, a soil-transmitted helminth (STH) that occurs worldwide, is probably the most neglected of all neglected tropical diseases (NTDs) [1–5]. *S*. *stercoralis* is endemic in humid and warm regions where sanitation and hygiene conditions are poor [2, 5, 6]. Its global prevalence is largely underestimated due to the use of inadequate diagnostic techniques. A rough (and probably still conservative) estimate of 220–370 million infection cases has recently been put forward, corresponding to half of the number of hookworm cases [4, 7]. Many epidemiological aspects of *S*. *stercoralis* infection are unknown or poorly documented, but data from recent studies suggest prevalence rates between 10% and 40%, and possibly up to 60%, in the tropics and subtropics [6].

*S*. *stercoralis* larvae living in soil contaminated by human faeces penetrate the intact human skin and eventually reach the small intestine. Walking barefoot or prolonged contact with contaminated soil when farming are known risk factors for infection in areas with poor sanitation facilities [8]. Although about half of all infections remain asymptomatic, chronic symptomatic strongyloidiasis commonly involves diarrhoea, abdominal pain, urticaria and the so-called “larva currens” [8–10]. A particularity of *S*. *stercoralis* is its ability to replicate within its host, known as “autoinfection”, which may lead to potentially long-lasting and perpetual infections and to potentially fatal dissemination of the parasite [11–14].

Control programs against soil-transmitted helminths target infections with *Ascaris lumbricoides*, *Trichuris trichiura* and hookworms. The mainstay of the WHO’s “preventive chemotherapy” strategy is regular chemotherapy with albendazole or mebendazole, either through targeted treatment of specific at-risk groups or by mass-drug administration to entire populations [15, 16]. Health education and sanitation improvement are also recommended because they contribute to reducing transmission and are necessary for sustainable control [17].

Mebendazole and albendazole have a suboptimal effect on *S*. *stercoralis* and require a long-term treatment schedule, which precludes their use for large-scale control of this parasite [18, 19]. Ivermectin is the drug of choice against *S*. *stercoralis* and a single oral dose has been shown to be highly efficacious [19–21].

To date, there is no control strategy against *S*. *stercoralis*. Current evidence calls for action, but key aspects of *S*. *stercoralis* infections need to be documented to assess the effectiveness of preventive chemotherapy and to adequately target control measures [1–4, 22, 23]. A major question is whether *S*. *stercoralis* control could be integrated into existing STH control programs.

The purpose of this large two-year cohort study was to assess the impact of ivermectin for chemotherapy-based control, to determine which age groups should be targeted and to identify risk factors for *S*. *stercoralis* incident infections among the general population in an endemic setting.

## Methods

### Ethics statement

Ethical approval was obtained from the National Ethics Committee for Health Research, Ministry of Health, Cambodia (NECHR, reference number 192, dated December 19, 2011) and from the Ethics Committee of Basel and Baselland, Switzerland (EKBB, reference number 18/12, dated February 23, 2012). All participants received an explanation of the study goals and procedures prior to enrollment. Written informed consent was obtained from all participating adults, while consent from participants aged 2–18 years was obtained from the parents or legal guardians.

### Study setting and population

The study was conducted among the general population of Preah Vihear, a rural province in Northern Cambodia with an estimated population of 234,370 in 2013 [24]. STH, including *S*. *stercoralis*, are highly endemic [22]. Eight villages that had not been previously exposed to *S*. *stercoralis* treatment were selected in the Rovieng district. In each village, all households were included and all household members over two years old were eligible.

### Study design and participants

This study was a two-year prospective intervention (cohort) study, consisting of a baseline survey conducted between February and June 2012 and two follow-up surveys of enrolled individuals, conducted after 12 months (January-March 2013) and 24 months (February-March 2014).

**Fig 1:**
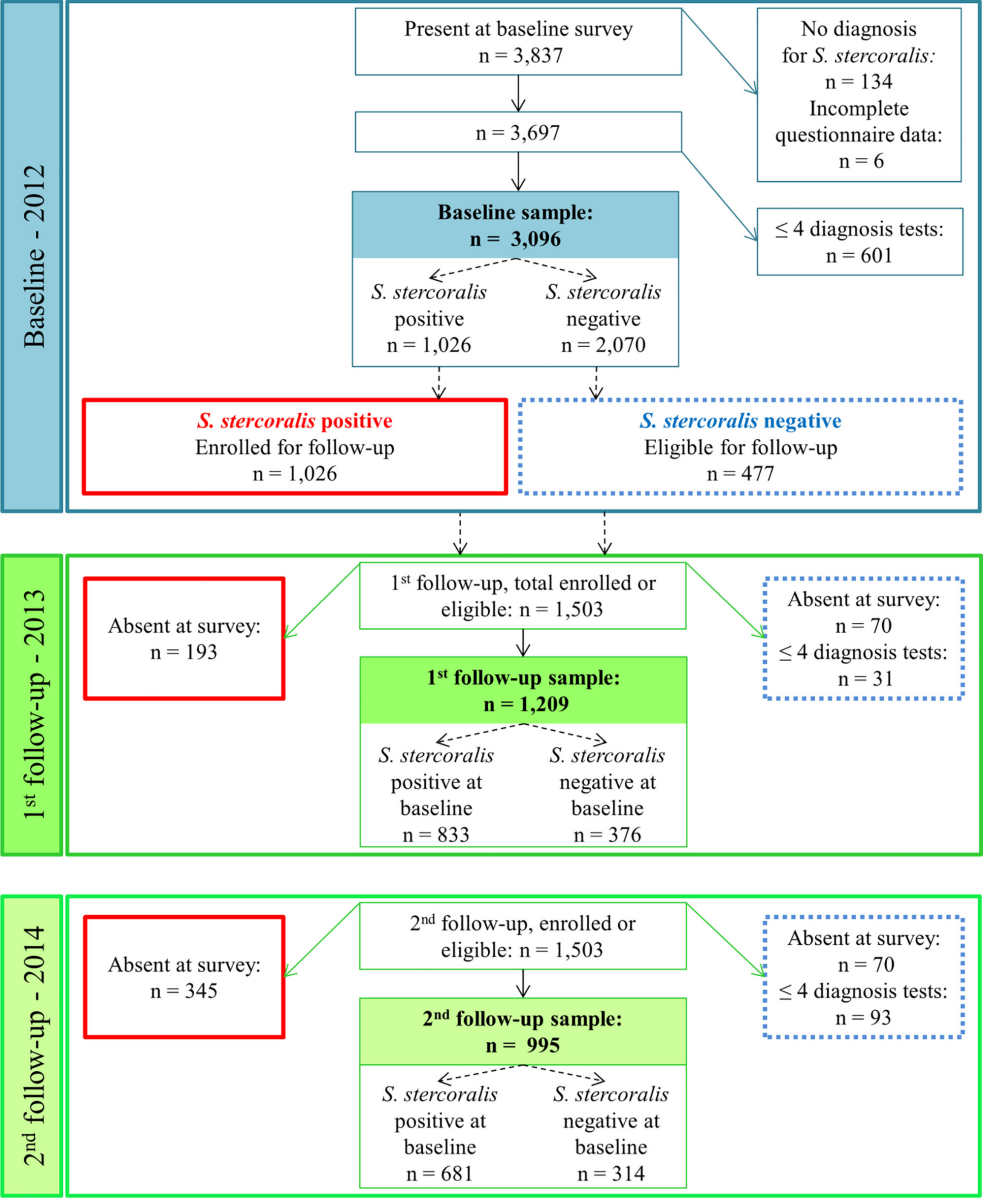
Study diagram. Flowchart detailing compliance levels and the number of participants included at baseline (2012) and at follow-up (2013 & 2014) among community members from eight villages in Preah Vihear province, Cambodia. Negative participants with one (out of four) or more missing diagnostic examinations were excluded from the analysis. Numbers in bold correspond to the size of the analyzed samples.

The study cohort included participants who submitted at least one stool sample and were either positive or negative at baseline. All *S*. *stercoralis* positive participants at baseline (2012) were enrolled. A subset of negative participants at baseline was asked to participate in the cohort and was selected as follows: in each of the eight villages, 18 to 26 households were randomly selected and visited again in 2013. Household members who were present and *S*. *stercoralis* negative at baseline (2012) were asked to participate in the two follow-up surveys. A study diagram is presented in Fig 1.

The intervention consisted of face-to-face health education on worm infections and hygiene and of administering a single oral dose of ivermectin (200μg/kg BW) to all *S*. *stercoralis* cases at baseline and follow-up. A sample of 290 *S*. *stercoralis*-infected participants at baseline was followed-up 21 days after treatment to estimate the cure rate. Other parasitic infections were treated according to the national guidelines [25].

### Demographic, socioeconomic, knowledge and behavioral data

Data on demographic features (age, sex, main occupation, level of education), knowledge about worms (sources of infection, health problems caused by worms) and hygiene practices (hand washing, shoe wearing, main defecation place) were obtained from each participant with a pre-tested questionnaire. Heads of households were interviewed about the size of the household, water and sanitation conditions, house construction material and ownership of household assets.

### Parasitological data

At each survey, two stool samples were collected on consecutive days from each participant. *S*. *stercoralis* was diagnosed using the Koga agar plate (KAP) culture [26] and the Baermann technique [27], performed on each sample. *S*. *stercoralis* larvae were identified through examination under a microscope and based on morphology. Combining those two methods on two samples has a 92.8% sensitivity rate [28]. A detailed description of this laboratory procedure is given elsewhere [22].

### Quality control

Technicians were specifically trained to identify *S*. *stercoralis* larvae. Throughout the study period, technicians were rigorously supervised by a qualified microscopist from the Swiss Tropical and Public Health Institute (Swiss TPH), Basel, Switzerland. Any unclear diagnosis was immediately discussed with both the qualified microscopist and the study supervisor.

### Statistical analysis

All (questionnaire and laboratory) data were double-entered and validated in EpiData version 3.1 (EpiData Association; Odense, Denmark). Data management and statistical analyses were performed in STATA version 13.0 (StataCorp LP; College Station, United States of America).

Two risk definitions of *S*. *stercoralis* infection were considered: the risk of *S*. *stercoralis* infection at baseline (prevalence) and the risk of *S*. *stercoralis* infection at follow-up (incidence). The latter included all cases occurring at any follow-up survey, either among participants found positive at baseline, treated and considered negative after treatment (“re-infection”) or participants diagnosed negative at baseline (”new-infection”).

A participant was considered *S*. *stercoralis* positive if at least one larva was found in any of the four samples or *S*. *stercoralis* negative if no larva was detected in the four samples. Participants with only negative results but with fewer than four analyzed samples were not included in the analysis. The risk of infection at follow-up was defined as a binary outcome, which took the value of one if a participant was infected at any of the follow-up surveys, regardless of their infection status at baseline, and zero otherwise. All reported results in this paper use the definitions described above, unless specified otherwise.

Age was centered at the mean of each sample (i.e. baseline or follow-up) and both squared and cubic terms were calculated. A socioeconomic index was built based on house construction material and asset ownership variables, using Principal Component Analysis (PCA) [29, 30]. Households were classified into wealth tertiles, with the first tertile corresponding to the least poor and the third tertile to the poorest. To ensure the comparability of household socio-economic status (SES) across time, the same asset weights were used to calculate the index of each year. Therefore, differences in SES between 2012 and 2014 relate to changes in the ownership of assets. For some of the subjects, age and education variables were inconsistent across the surveys. To resolve inconsistencies, the following procedure was used: if two of the three values were consistent then the third one was corrected so as to achieve consistency across all three values. Pearson’s chi-squared (χ^2^) test was used to compare proportions. The cure rate achieved by ivermectin was assumed to be the proportion of *S*. *stercoralis* patients who had four negative diagnosis results (Baermann method and Koga agar plate tests on two consecutive days) 21 days after treatment.

Mixed-effects logistic regression models were used to investigate the association of each risk definition with explanatory demographic, socioeconomic and behavioral variables, i.e. *S*. *stercoralis* infection risk at baseline (“baseline model”) and infection risk at follow-up (“follow-up model”). Village-level correlation was taken into account with a village-level random effect in both models. For the follow-up model, within-individual correlation was accounted for using an individual-level random effect.

The model building process was similar for the two models. Age and sex were not submitted for variable selection. The same set of demographic, socioeconomic, water and sanitation, and behavioral variables was subjected to selection for each outcome, with the exception of village-level prevalence at baseline and an indicator for baseline infection status that were used only in the follow-up model. Those variables are presented in S1 Table. For the follow-up model, age, occupation and level of education as of the previous year were used as explanatory variables. For behavior and knowledge, current year values were used because health education was always delivered after administering questionnaires and current values were also deemed more representative of the knowledge and behavior of the past months than the values of the previous survey.

Variable selection was first performed using mixed-effects bivariate logistic regressions, including the appropriate random effects as described above. Variables exhibiting an association at a significance level of 15% in the likelihood ratio test (LRT) were included in the multivariate logistic regression models. In case two explanatory variables were strongly correlated, only one of them was included in the model and this variable was selected based on the Akaike Information Criterion (AIC) of the resulting model.

Additionally, sex was checked to determine if it could be an effect modifier of any other variable in the baseline and follow-up models. For the follow-up model, the interaction between defecation place and occupation was checked, as well as whether infection status at baseline could be an effect modifier of any other variable in the model, in case infection status at baseline involved different risk factors at follow-up. Finally, the significance of the village-level random effect was assessed and the random effect was removed if the AIC indicated a better fit in absence of the random effect. We also conducted a mixed Poisson regression analysis providing incidence rate ratios instead of odds ratios [31].

To illustrate the impact of village-level sanitation coverage, the STATA command “margins” was used to predict the risk of infection at follow-up as a function of village-level sanitation coverage, while adjusting for all other covariates of the underlying risk factor model.

## Results

### Study population and compliance

At baseline, 3,837 participants were present and 3,697 had complete questionnaire data and at least one available diagnostic result for *S*. *stercoralis*, i.e. with either Baermann and/or KAP results available for one day (Fig 1).

All 1,026 *S*. *stercoralis* positive participants were enrolled and 477 *S*. *stercoralis* negative participants were eligible for enrollment. The compliance rate was 86.7% among the 1,503 eligible cohort participants. There were no differences in the proportions of males and females or in reported defecation place between compliant and non-compliant participants at baseline. The proportion of preschool-aged children and of adults who declared staying at home, having a small business or working in the tertiary sector were higher in the non-compliant group, while participants who attained primary school were better represented in the compliant group. Therefore, these variables were adjusted for in the risk factor analysis.

According to the outcome definition adopted in this work, samples from 3,096 participants at baseline were analyzed, while the study cohort included 1,269 participants, of whom 873 were positive and 396 were negative at baseline. For the follow-up, 935 cohort participants were present at both surveys, 274 were present only at the first follow-up and 60 were present only at the second follow-up. Hence, 1,269 participants were included in the analysis of infection risk at follow-up, i.e. all participants who were present at one or both follow-up surveys. Fig 1 displays compliance levels and the number of participants excluded from the analysis at each survey. The characteristics of the baseline and cohort participants are given in S1 Table. Overall, the cohort appeared to be representative of the baseline sample, with similar distributions of covariates. The only notable difference between the baseline and cohort samples was the frequency of females, which was higher at baseline (n = 1,702, 55.0%) while more males were represented in the cohort (n = 653, 51.4%). This difference is due to the higher risk of infection among males and was statistically significant (χ^2^ = 14.9, p<0.001). Cohort participants were older than non-participants (median = 23 vs. 25 years) at baseline. The most frequent occupation was rice farmer (baseline: 50.5%; cohort: 54.4%). About half (baseline: 55.9%; cohort: 55.1%) of participants did not have access to sanitation and 57.9% of participants declared regularly defecating in the open at baseline. Almost all existing sanitation facilities were latrines types recognized as efficient in preventing from exposure to excreta, with 93% of them being ventilated improved pit latrines (96%) or pour-flush toilets. No village had full sanitation coverage and the village-level proportion of households owning a latrine ranged from 4.2% to 77.6%.

### *S*. *stercoralis* infection risk at baseline and at follow-up

**Fig 2:**
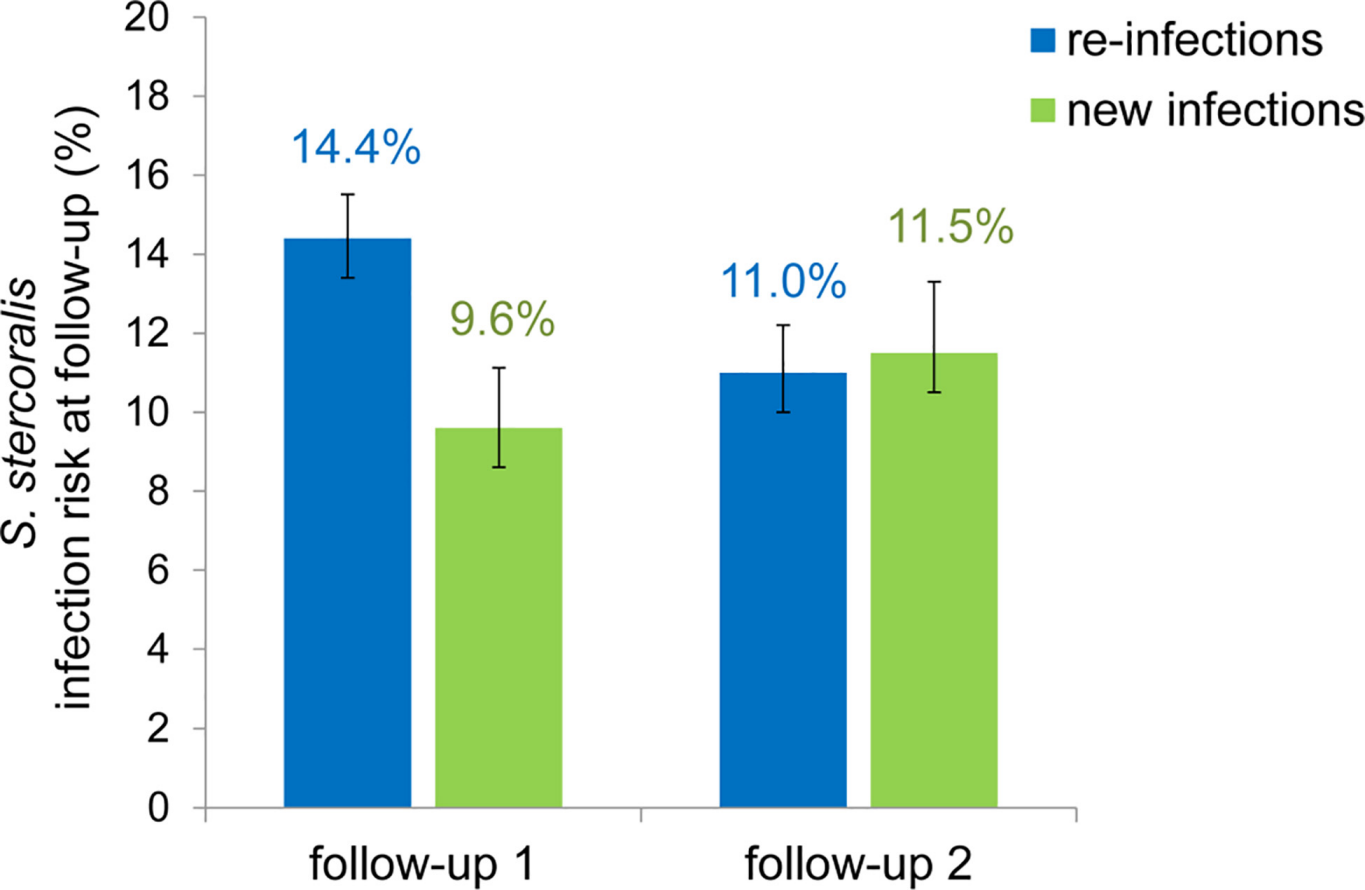
Rates of *S*. *stercoralis* infection at follow-up surveys among participants who tested positive or negative at baseline. Re-infections: *S*. *stercoralis* infection at follow-up among participants who tested positive at baseline. New infections: *S*. *stercoralis* infection at follow-up among participants who tested negative at baseline. Error bars indicate 95% confidence interval. Data were obtained from repeated surveys carried out among 1,269 participants at follow-up in eight villages of Preah Vihear province, Cambodia, in 2013 (follow-up 1) and 2014 (follow-up 2).

The prevalence of *S*. *stercoralis* infection at baseline was 33.1%, (95% Confidence Interval (CI): 31.5–34.8). Prevalence was similar across the eight study villages (χ^2^ = 10.62, p = 0.16) and ranged from 24.2% (95%CI: 19.7–29.2) to 33.8% (95%CI: 27.6–40.4). Among the cohort participants who were infected at baseline, 120/833 and 75/681 participants were found to have been re-infected, yielding re-infection rates of 14.4% (95%CI: 12.1–17.0) and 11.0% (95%CI: 8.6–13.6) at the first (2013) and second (2014) follow-up, respectively. Among the cohort participants who were infection-free at baseline, 36/376 and 36/314 were found to have been infected at the first and second follow-up, respectively, resulting in new infection rates of 9.6% (95%CI: 6.8–13.0) and 11.5% (95%CI: 8.1–15.5) in 2013 and 2014, respectively. Re-infection rates significantly varied across villages (χ^2^ = 32.2, p<0.001) and ranged from 8.3% (95%CI: 3.1–17.3) to 20.1% (15.5–25.3). Compared to the positive cohort, the rate of infection was significantly lower among the negative cohort at the first follow-up, but there was no difference at the second follow-up. Fig 2 displays the rates of *S*. *stercoralis* infection among participants testing positive or negative at baseline, at each follow-up.

### Cure rate of ivermectin

Of the 290 participants who were enrolled for follow-up 21 days after treatment, 261 (90.0%) were present at follow-up and of those, 206 (78.9%) had complete diagnostic testing (i.e. four results regardless of the infection status). 7/206 patients had not been cured, so the cure rate achieved by ivermectin was 96.6% (95%CI: 93.1–98.6).

### Risk factors for *S*. *stercoralis* infection at baseline and at follow-up

The results of the bivariate mixed-effects logistic regression models at baseline (prevalent cases) and follow-up (incident cases) are presented in S2 Table. At baseline, the village-level random effect was not significant in any bivariate model, so risk factors for infection at baseline were explored using a simple logistic regression model. At follow-up, the village-level random effect lost significance upon introduction of the proportion of households owning a latrine in each village, which suggests that this variable accounted for most of the between-village differences in infection risk.

The results of the multivariate logistic regression models built for baseline and follow-up are presented in Table 1. No interaction was found either in the baseline or follow-up model. At baseline, females had lower odds of being infected and infection risk increased with age. The poorest were at a higher risk for infection, as were participants with a higher education level. At follow-up, the risk of acquiring a new infection decreased with increasing sanitation coverage at village level. Neither age nor sex was significantly associated with the risk of acquiring an *S*. *stercoralis* infection at follow-up. Rice farmers had higher odds of acquiring an infection, as did participants who reported regularly defecating in rice fields or water. Another behavioral risk factor was not wearing shoes, both at home and when going to the toilet. The odds ratios obtained with this multivariate model were similar to risk ratios produced by the mixed Poisson multivariate model. Incidence rate ratios obtained from this model are presented in S3 Table.

**Table 1:** Risk factors for infection with *S*. *stercoralis* at baseline and follow-up.

		Infection risk at baseline			Infection risk at follow-up		
		OR	95% CI	p-value	OR	95% CI	p-value
Variable	Category						
Sex	Male	1.00	-	-	1.00	-	-
	Female	**0.46**	0.40–0.54	< 0.001	0.80	0.61–1.04	0.096
Age (years)	Linear term	**1.02**	1.01–1.03	< 0.001	0.99	0.98–1.00	0.094
	Quadratic term	0.99	0.99–1.00	0.060	n.a.	n.a.	n.a.
Level of education	Primary	1.00	-	-	1.00	-	-
	No schooling	0.85	0.64–1.14	0.281	**0.47**	0.27–0.84	0.010
	Secondary or higher	**1.29**	1.07–1.56	0.007	1.15	0.86–1.55	0.335
Socioeconomic level	Least poor				n.a.	n.a.	n.a.
	Poor	1.14	0.94–1.37	0.184	n.a.	n.a.	n.a.
	Poorest	**1.37**	1.12–1.68	0.003	n.a.	n.a.	n.a.
Occupation	School, at home, other	1.00	-	-	1.00	-	-
	Rice farmer	1.12	0.86–1.45	0.403	**1.60**	1.10–2.31	0.013
Reported regular place of defecation	Toilet	1.00	-	-	1.00	-	-
	Forest	1.14	0.93–1.40	0.207	1.11	0.79–1.56	0.559
	Rice field or water	0.95	0.78–1.16	0.605	**1.53**	1.09–2.14	0.014
	Behind the house	0.88	0.62–1.26	0.486	1.11	0.60–2.08	0.738
Wearing shoes, frequency	Often	1.00	-	-	n.a.	n.a.	n.a.
	Always	1.08	0.90–1.28	0.409	n.a.	n.a.	n.a.
	Sometimes or never	1.14	0.83–1.56	0.430	n.a.	n.a.	n.a.
Wearing shoes, at home and/or to toilets	Any other case	n.a.	n.a.	n.a.	1.00	-	-
	No at home, yes to toilets	n.a.	n.a.	n.a.	2.14	0.69–6.63	0.189
	No at home, no to toilets				**3.20**	1.38–7.42	0.007
Washing hands after defecating	Yes	n.a.	n.a.	n.a.	1.00		
	No	n.a.	n.a.	n.a.	1.42	0.94–2.14	0.094
Do you know anything about worms?	No	1.00	-	-	n.a.	n.a.	n.a.
	Yes	0.94	0.78–1.14	0.552	n.a.	n.a.	n.a.
Positive for *S*. *stercoralis* at baseline	No	n.a.	n.a.	n.a.	1.00	-	-
	Yes	n.a.	n.a.	n.a.	1.17	0.87–1.57	0.301
Proportion of houses with latrines in the village (%)		n.a.	n.a.	n.a.	**0.989**	0.982–0.996	0.003

OR: odds ratio

CI: confidence interval

OR in bold are significant at 5% level.

Data were obtained from a two-year cohort survey carried out among 3,096 participants at baseline (2012) and 1,269 participants at follow-up (2013 & 2014), in eight villages of Preah Vihear province, Cambodia.

**Fig 3:**
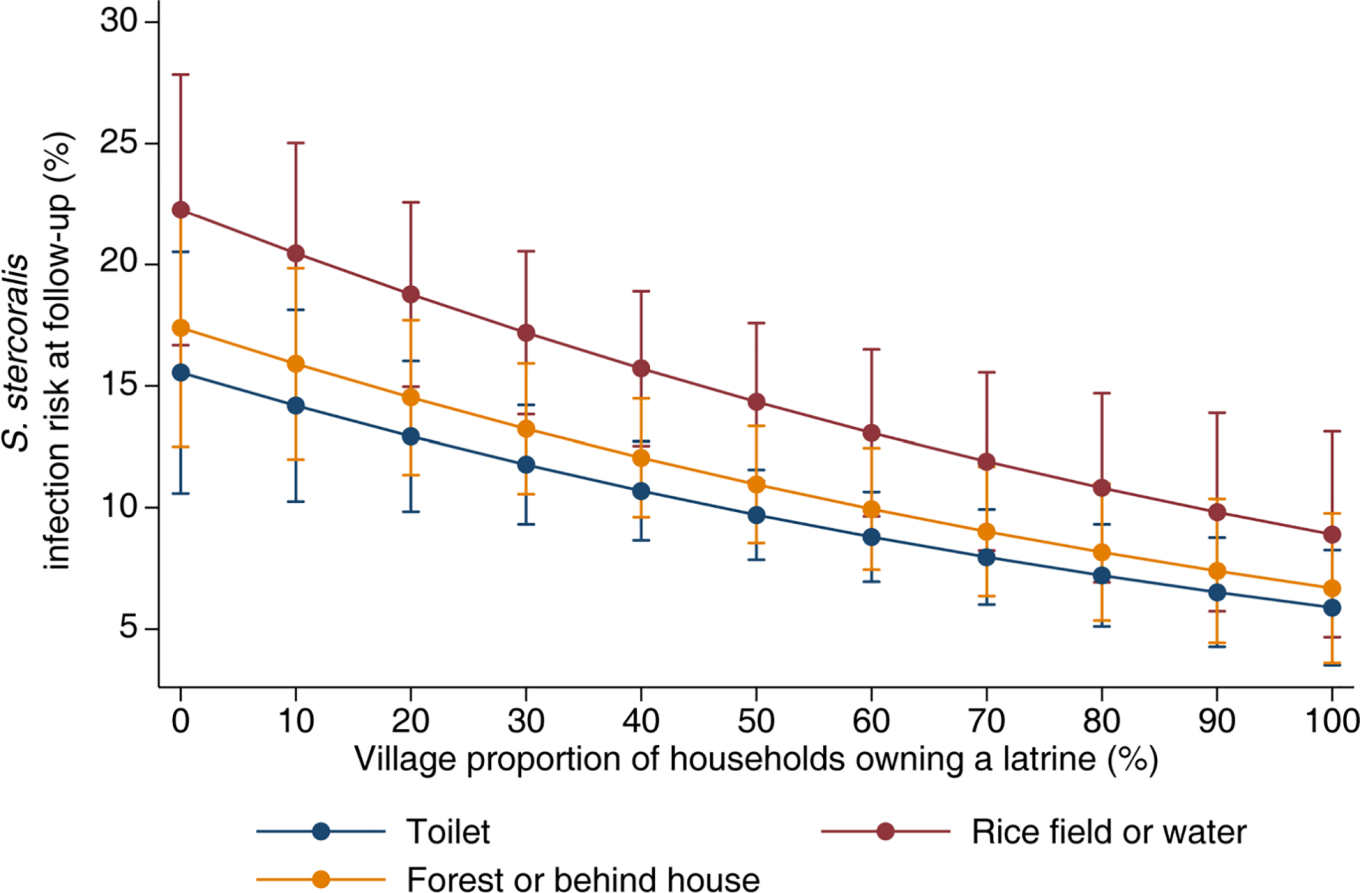
Predicted risk of *S*. *stercoralis* infection risk at follow-up by village sanitation coverage level and defecation place. Data were obtained from longitudinal surveys carried out among 1,269 participants at follow-up in eight villages of Preah Vihear province, Cambodia, between 2013 and 2014. The prediction was adjusted for sex, age, level of education, occupation, shoe wearing, hand washing after defecating and infection status at baseline.

Fig 3 shows the predicted risk of *S*. *stercoralis* infection at follow-up as a function of the village-level proportion of households owning a latrine and reported individual defecation place. The risk of *S*. *stercoralis* infection at follow-up decreases with increasing sanitation coverage as expressed by the village-level proportion of households owning improved latrines. While the risk of individuals regularly defecating in rice fields or water is the highest, the risk of acquiring a *S*. *stercoralis* infection at follow-up for individuals usually defecating in toilets *vs*. in the forest or behind the house appear similar and reflect the absence of a significant protective effect of defecation in latrines, at individual level.

## Discussion

This two-year community-based cohort study documents, for the first time to our knowledge, incidence rates and risk factors for incidence of *S*. *stercoralis* among the general population and provides essential information to guide control efforts: ivermectin chemotherapy against *S*. *stercoralis* infections is highly beneficial, and its impact is enhanced by community-level improved sanitation coverage.

About one in three of the 3,000 participants present at baseline were infected with *S*. *stercoralis*. A cohort of 1,269 participants was followed-up over two years and *S*. *stercoralis* re-infection rates were 14% and 11% at the first and second follow-up, respectively. The rates of newly acquired infections among participants who were negative at baseline were 10% and 11% in 2013 and 2014, respectively. The re-infection rates estimated here confirm results by Khieu and colleagues who found a re-infection rate of 31% in a 2-year cohort study of 300 schoolchildren in semi-rural Cambodia [32]. Since only eight individuals were positive at both follow-ups, a rate of similar magnitude might have been observed after two years if cases found at the first follow-up had not been treated.

Most importantly, the *S*. *stercoralis* infection risk at follow-up was low, with almost 90% of cohort participants testing negative one year after treatment or after having been diagnosed negative at baseline, indicating that populations strongly benefited from ivermectin treatment. Indeed, an incidence rate of around 13% is particularly low, even compared to that of hookworm, which has the lowest re-infection rates of the three other STH [33].

A striking finding of the present work was the corresponding decrease of *S*. *stercoralis* infection risk at follow-up with increasing sanitation coverage measured at community level. While the rationale behind improving sanitation for STH control is to prevent re-infection by decreasing transmission, evidence supporting this fact remains rare and mostly arises from cross-sectional studies using latrine availability or use at individual level.

We did not find any protective effect of defecating in latrines on *S*. *stercoralis* infection risk at baseline or follow-up. The association between *S*. *stercoralis* infections and sanitation has rarely been studied so far. Varying results were obtained mostly from cross-sectional studies, which either found no association or a decreased risk associated with defecation in or access to improved latrines [22, 34–36]. In our study, we found a strong impact of sanitation coverage in combination with treatment which explained the differences in infection rates at follow-up across villages. Two key aspects underlie this finding. First, coverage was strongly correlated with use in this setting, with almost all participants living in a house equipped with latrines declaring regularly defecating in them. Second, more than 90% of existing sanitation facilities were improved latrines, which effectively prevent environmental contamination [37, 38]. This result is in line with another study that also found a protective effect of 75% sanitation coverage and above on STH infection [39].

Unlike infection risk at follow-up, baseline prevalence was similar across villages and was not associated with sanitation coverage. This suggests that, in absence of treatment, the protective effect of sanitation coverage levels-off with time, with parasites steadily accumulating in the environment. In that case, the association between infection risk and sanitation coverage and use may not be observable in the absence of a longitudinal approach. Additionally, participants who declared not wearing shoes, both at home and when going to defecate, had a three-fold higher risk of acquiring an infection by the time of follow-up. This result adequately reflects that infection occurs due to exposure of bare feet to larvae and is in line with meta-analysis findings showing that the risk of infection with *S*. *stercoralis* was almost halved by footwear use (OR: 0.56, 95%CI: 0.38–0.83) [40].

It is widely accepted by the STH control community that water, sanitation and hygiene (WASH) improvement is important for controlling and preventing those infections [41–43]. However, sanitation measures have not concretely been included in STH intervention packages because implementing them is particularly challenging due to their high cost, complexity, need for cross-sectorial collaboration, and lack of perceived need by communities [41, 42]. Regarding the latter, promoting behavior change and/or triggering demand for sanitation through initiatives like community-led total sanitation (CLTS) and targeted social marketing or social networking appears to be useful but actual evidence of their impact is scarce [42, 44–46]. There is still a need to assess and quantify which sanitation improvement measures are effective in reducing *S*. *stercoralis* (as well as other STH) transmission, including combinations with chemotherapy, and according to which ecological and socio-cultural settings.

Infection at follow-up was not associated with age, so no age risk group could be identified as a target for control. Given the implications of this result, various additional models were run throughout the model building process to test the relationship between infection risk at follow-up and age. None provided evidence in favor of a significant role of age, indicating that the result presented here is robust. Yet, *S*. *stercoralis* prevalence at baseline did increase with age, a result consistent with other findings from all continents [5, 9, 22, 35, 47]. Combined, the relationships between age and prevalence or incidence indicate that *S*. *stercoralis* infections are permanently acquired through life and that higher prevalence rates observed among adults result from infections that are maintained and accumulated over time [22].

Finally, regarding other risk factors, farmers were found to have higher odds of acquiring an infection, which was reflected by the positive association between infection risk at follow-up and both occupation and defecating in rice fields. This result is consistent with farmers’ frequent and intense contact with soil and is in line with the fact that *S*. *stercoralis* infection is known to be an occupational disease of farmers and miners, even in temperate climates [6].

Our study has some limitations. First, we restricted the inclusion of *S*. *stercoralis* negative participants to those who had four negative diagnostic results to ensure a high specificity while keeping the maximum number of positive observations in the sample. This approach resulted in a slight overestimation of prevalence at baseline, i.e. 33.1% *vs*. 29.3% with a complete case analysis, but it did not significantly affect the incidence rates. Moreover, because *S*. *stercoralis* can replicate within its host, an undetected infection can reappear through auto-infection [11]. This may have led to overestimation of the ivermectin cure rate and infection risk at follow-up. Given the high cure rate achieved by ivermectin and the rapid clearance of parasites after a single oral dose, the number of re-emerging infections and uncured patients should be low. Nonetheless, the genotyping of parasites before and after treatment is needed to assess the proportion of re-emerging *vs*. new infections, which is an important aspect of *S*. *stercoralis* infection [32, 48]. The cure rate achieved by ivermectin has likely been overestimated due to the use of coprological methods, but this issue might have been mitigated by the highly sensitive diagnostic approach used in this study [3, 49]. An alternative way to confirm cure would be to use serological tests which would involve measuring antibody titers 6–12 months after treatment but which would be inappropriate in a high risk setting where reinfection occurs [50, 51]. Second, one village had a very low proportion of households owning a latrine (4%), which could have biased the association with follow-up infection risk. However, the association remained significant after excluding this village from the analysis (the results from this model are available in S4 Table). Additionally, a more flexible model (i.e. including a quadratic term for sanitation coverage) was run to assess the potential non-linearity of this effect. Although this term was not significant, the resulting dose-response curve suggested that the effect of sanitation coverage of *S*. *stercoralis* might level off at around 60%. The existence of such a threshold would be consistent with other findings [39, 52]. This aspect might be of importance when setting goals for sanitation improvement measures. While we are confident in our results about sanitation coverage, additional studies including a larger number of villages could help to assess whether there is indeed a threshold. The possibility that sanitation acted in a fashion similar to herd immunity as suggested by our results, would have far-reaching implications in terms of equity, since even partial sanitation coverage would benefit entire communities, including those who cannot afford latrines. Finally, the generalization of findings presented here will necessitate additional studies and modeling across different settings to account for variation in geographic and living conditions, including access to improved sanitation across regions, when assessing the impact of both treatment and sanitation on *S*. *stercoralis* infections [53].

The high efficacy of ivermectin and the low incidence rates estimated in the present work suggest that mass ivermectin chemotherapy as a control measure against *S*. *stercoralis* would have a strong impact. Our results actually confirm recent findings from a retrospective study conducted in Ecuador which found that mass drug administration of ivermectin targeting onchocerciasis had a significant impact on *S*. *stercoralis* prevalence [54]. Whether treatment should be delivered annually or more or less frequently cannot be addressed by the present study design, but a cost-effectiveness analysis could help to assess this component of control. In the absence of age-specific morbidity data for chronic infections and because the risk of developing hyper-infection is similar at any age, the similarity of incident infection risk across ages and the highest prevalence rates among adults in populations naïve to treatment suggest a community-wide approach.

Reaching entire communities may raise feasibility and treatment availability issues, although the experience of lymphatic filariasis and onchocerciasis show that community-wide mass treatment is feasible [55]. For Cambodia, this should not be a major issue given its effective and well-established delivery systems of treatment against other STH, working through health centres with community health workers and, lately, even in factories [56–58]. The affordability of treatment is of greater concern. Ivermectin is not subsidized or donated in Cambodia, where a tablet produced by a certified good manufacturing practice company is available for Cambodia at 10 USD and two to four tablets are needed to treat one individual. Unfortunately, and whatever the target population, such a cost currently precludes the implementation of large-scale *S*. *stercoralis* control. Although the difficulties of improving sanitation are widely acknowledged, cost-effectiveness studies for sanitation measures might be of interest in the framework of *S*. *stercoralis* control in Cambodia, given the current high cost of ivermectin and the potential impact of sanitation measures on other STH and diarrhoeal diseases. Additional research on sanitation impact should include assessments of latrine promotion and behavior change measures.

The findings presented here indicate that *S*. *stercoralis* should be integrated into existing STH control programs and include adults. In addition, improved sanitation enhances the effect of successful treatment by reducing *S*. *stercoralis* transmission, so sanitation improvement measures using new participatory approaches such as CLTS or social marketing should be considered [59, 60]. However controlling *S*. *stercoralis* will not be achievable unless funds are made available or generic ivermectin is produced at an affordable price so that low- and middle-income countries, which are the most affected, can start tackling the *S*. *stercoralis* problem.

## Supporting Information

S1 Checklist STROBE statement checklist. (PDF)

S1 Table Baseline characteristics of participants included in the analysis of *S*. *stercoralis* infection at baseline and at follow-up. IQR: interquartile range. The baseline sample (left column) includes both participants and non-participants in the cohort. The cohort sample includes all cohort participants regardless of whether they were present at one or two follow-up survey(s). ^a^ For age, in case of inconsistencies in the age across years, if two of the three values were consistent then the third one was corrected so as to achieve consistency across all three values. ^b^ For education attainment, the same procedure as for age was used in case of inconsistencies reported by individuals over 20 years old and not attending school anymore. Data were obtained from a two-year cohort survey carried out among 3,096 participants at baseline (2012) and 1,269 participants at follow-up (2013 & 2014), in eight villages of Preah Vihear province, Cambodia.
(PDF)

S2 Table Bivariate associations between explanatory variables submitted for variable selection and *S*. *stercoralis* infection risk at baseline and at follow-up. OR: odds ratio; CI: confidence interval; LRT: likelihood ratio test. ^a^: For infection risk at follow-up, the values are that of the previous year, i.e. 2012 for the first follow-up and 2013 for the second follow-up. ^b^: For infection risk at follow-up, the baseline values of socioeconomic level were used. Data were obtained from a two-year cohort survey carried out among 3,096 participants at baseline (2012) and 1,269 participants at follow-up (2013 & 2014), in eight villages of Preah Vihear province, Cambodia.
(PDF)

S3 Table Incidence rate ratios for risk factors of *S*. *stercoralis* infection risk at follow-up. IRR: incidence rate ratio; CI: confidence interval; IRR in bold are significant at 5% level. As Poisson regression provides biased standard errors when being applied to binary data, confidence intervals were computed using Miettinen's formula IRR^(1 +/- 1.96/t) where t is the valid t-value of the respective parameter estimate in the corresponding mixed logistic regression model.
(PDF)

S4 Table Results of the multivariate model for *S*. *stercoralis* infection risk at follow-up, excluding the village with 4% sanitation coverage. OR: odds ratio; CI: confidence interval; OR in bold are significant at 5% level. Data were obtained from a two-year cohort survey carried out among 1,128 participants at follow-up (2013 & 2014), in seven villages of Preah Vihear province, Cambodia.
(PDF)
