# Supplementary material for: Ivermectin Treatment and Sanitation Effectively Reduce Strongyloides stercoralis Infection Risk in Rural Communities in Cambodia
Source: PLoS Negl Trop Dis. 2016 Aug 22;10(8):e0004909. doi: 10.1371/journal.pntd.0004909 (PMC4993485; doi:10.1371/journal.pntd.0004909)
Supplement: S1 Checklist — (PDF) [file pntd.0004909.s001.pdf]

S1. STROBE Statement checklist

	Item No	Recommendation
**Title and abstract**	1	(*a*) Indicate the study's design with a commonly used term in the title or the abstract<br>Page 1- in the abstract
		(*b*) Provide in the abstract an informative and balanced summary of what was done and what was found<br>Page 1
**Introduction**		
Background/rationale	2	Explain the scientific background and rationale for the investigation being reported<br>Page 2-3
Objectives	3	State specific objectives, including any prespecified hypotheses<br>Page 3 – last paragraph of the introduction
**Methods**		
Study design	4	Present key elements of study design early in the paper<br>Page 4 – Methods, Section "Study design and participants"
Setting	5	Describe the setting, locations, and relevant dates, including periods of recruitment, exposure, follow-up, and data collection<br>Page 4, Methods, Sections "Study setting and population" and "Study design and participants"
Participants	6	(*a*) *Cross-sectional study*—Give the eligibility criteria, and the sources and methods of selection of participants<br>Serial cross-sectional. Page 4, Methods, Section and "Study design and participants"
		(*b*) *Cohort study*—For matched studies, give matching criteria and number of exposed and unexposed – n.a.<br>*Case-control study*—For matched studies, give matching criteria and the number of controls per case – n.a.
Variables	7	Clearly define all outcomes, exposures, predictors, potential confounders, and effect modifiers. Give diagnostic criteria, if applicable<br>Outcomes: Methods, page 6, Section statistical analysis, § 2&3<br>Exposures, predictors, potential confounders, and effect modifiers: Methods, page 6, Section "Demographic, socioeconomic, knowledge and behavioural data; Section statistical analysis, § 4, 7; Supplementary table S1<br>Diagnosis: Methods, page 5 , Section Parasitological data
Data sources/ measurement	8*	For each variable of interest, give sources of data and details of methods of assessment (measurement).<br>Sections "Demographic, socioeconomic, knowledge and behavioural data", "Parasitological data"<br>In all groups, infection and exposure measurements were done with the same methodology.
Bias	9	Describe any efforts to address potential sources of bias<br>Methods, page 8, Section "statistical analysis", § 5, 7, 8; results Section "Study population and compliance" §2.
Study size	10	Explain how the study size was arrived at<br>Methods, section "Study setting and population", Section "Study design and participants"; Figure 1.

Quantitative variables	11	Explain how quantitative variables were handled in the analyses. If applicable, describe which groupings were chosen and why<br>Methods, page 6-7, Section statistical analysis, §4.
Statistical methods	12	(*a*) Describe all statistical methods, including those used to control for confounding<br>Methods, page 6-8, Section statistical analysis, §8
		(*b*) Describe any methods used to examine subgroups and interactions<br>Methods, page 8, Section statistical analysis, §4 to 7
		(*c*) Explain how missing data were addressed<br>Methods, section "Statistical analysis" §2<br>Results, section "Study population and compliance", §2<br>For explanatory variables, only 6 participants had missing questionnaire data. They were excluded from the analysis (c.f. Figure 1).
		(*d*) *Cohort study*—If applicable, explain how loss to follow-up was addressed<br>*Case-control study*—If applicable, explain how matching of cases and controls was addressed<br>*Cross-sectional study*—If applicable, describe analytical methods taking account of sampling strategy
		(*e*) Describe any sensitivity analyses<br>Several models were run to assess the role of age. NOT SHOWN in paper. IS it OK?<br>Discussion § 6 & 7 => several models were run to assess the robustness of the results obtained for age and for village-level sanitation coverage. There are only mentioned in the discussion since those models are not present in the paper.
**Results**		
Participants	13*	(a) Report numbers of individuals at each stage of study—eg numbers potentially eligible, examined for eligibility, confirmed eligible, included in the study, completing follow-up, and analysed<br>Results, page 9, §1 to 3
		(b) Give reasons for non-participation at each stage<br>Results, page 9, §2
		(c) Consider use of a flow diagram<br>Figure 1
Descriptive data	14*	(a) Give characteristics of study participants (eg demographic, clinical, social) and information on exposures and potential confounders<br>Supplementary table S1
		(b) Indicate number of participants with missing data for each variable of interest<br>Results Section "Study population and compliance", Figure 1.
		(c) *Cohort study*—Summarise follow-up time (eg, average and total amount)<br>Section "Study population and compliance" §3
Outcome data	15*	*Cohort study*—Report numbers of outcome events or summary measures over time<br>Section *S. stercoralis* infection risk at baseline and follow-up
		Case-control study—Report numbers in each exposure category, or summary measures of exposure
		Cross-sectional study—Report numbers of outcome events or summary measures
Main results	16	(*a*) Give unadjusted estimates and, if applicable, confounder-adjusted estimates and their precision (eg, 95% confidence interval). Make clear which confounders were adjusted for and why they were included<br>Unadjusted estimates: Supplementary table 2

		Confounder-adjusted estimates: Table 1
		(*b*) Report category boundaries when continuous variables were categorized<br>n.a.
		(*c*) If relevant, consider translating estimates of relative risk into absolute risk for a meaningful time period<br>n.a.
Other analyses	17	Report other analyses done—eg analyses of subgroups and interactions, and sensitivity analyses<br>Section "Risk factors for S. stercoralis infection at baseline and follow-up", § 2
**Discussion**		
Key results	18	Summarise key results with reference to study objectives<br>§ 1 to 3
Limitations	19	Discuss limitations of the study, taking into account sources of potential bias or imprecision. Discuss both direction and magnitude of any potential bias<br>§7
Interpretation	20	Give a cautious overall interpretation of results considering objectives, limitations, multiplicity of analyses, results from similar studies, and other relevant evidence<br>Done, all across discussion
Generalisability	21	Discuss the generalisability (external validity) of the study results<br>§7
**Other information**		
Funding	22	Give the source of funding and the role of the funders for the present study and, if applicable, for the original study on which the present article is based<br>Abstract

*Give information separately for cases and controls in case-control studies and, if applicable, for exposed and unexposed groups in cohort and cross-sectional studies.

Note: An Explanation and Elaboration article discusses each checklist item and gives methodological background and published examples of transparent reporting. The STROBE checklist is best used in conjunction with this article (freely available on the Web sites of PLoS Medicine at http://www.plosmedicine.org/, Annals of Internal Medicine at http://www.annals.org/, and Epidemiology at http://www.epidem.com/). Information on the STROBE Initiative is available at www.strobe-statement.org.
