# Supplementary material for: Ivermectin Treatment and Sanitation Effectively Reduce Strongyloides stercoralis Infection Risk in Rural Communities in Cambodia
Source: PLoS Negl Trop Dis. 2016 Aug 22;10(8):e0004909. doi: 10.1371/journal.pntd.0004909 (PMC4993485; doi:10.1371/journal.pntd.0004909)
Supplement: S1 Table — IQR: interquartile range. The baseline sample (left column) includes both participants and non-participants in the cohort. The cohort sample includes all cohort participants regardless of whether they were present at one or two follow-up survey(s). a For age, in case of inconsistencies in the age across years, if two of the three values were consistent then the third one was corrected so as to achieve consistency across all three values. b For education attainment, the same procedure as for age was used in case of inconsistencies reported by individuals over 20 years old and not attending school anymore. Data were obtained from a two-year cohort survey carried out among 3,096 participants at baseline (2012) and 1,269 participants at follow-up (2013 & 2014), in eight villages of Preah Vihear province, Cambodia. (PDF) [file pntd.0004909.s002.pdf]

S1 Table. Baseline characteristics of participants included in the analysis of *S. stercoralis* infection at baseline and at follow-up

		**Baseline**	**Cohort**
		(N = 3,096)	(N=1,269)
**Variable**		**Median; IQR**	**Median; IQR**
Age (years) [a]		23 ; 26	25; 28
	**Category**	**n (%)**	**n (%)**
Sex	Male	1,394 (45.0)	653 (51.5)
	Female	1,702 (55.0)	616 (48.5)
Level of education [b]	No schooling	449 (14.5)	169 (13.3)
	Primary	1,700 (54.9)	718 (56.6)
	Secondary and higher	947 (30.6)	382 (30.1)
Occupation	Rice farmer	1,564 (50.5)	690 (54.4)
	At home	331 (10.7)	123 (9.7)
	School	1,085 (35.0)	407 (32.1)
	Tertiary, business, other	116 (3.8)	49 (3.8)
Reported regular place of defecation	Toilet	1,326 (42.8)	539 (42.5)
	Forest	685 (22.1)	306 (24.1)
	Rice field or water	857 (27.7)	247 (27.3)
	Behind house	228 (7.4)	77 (6.1)
Availability of toilets at home	No	1,730 (55.9)	700 (55.2)
	Yes	1,366 (44.1)	569 (44.8)
Wearing shoes, frequency	Always	1,522 (49.2)	459 (36.2)
	Often	1,174 (37.9)	661 (52.1)
	Sometimes or never	400 (12.9)	149 (11.7)
Wearing shoes at work or school	Yes	2,874 (92.8)	1,187 (93.5)
	No	222 (7.2)	82 (6.5)
Wearing shoes at home	Yes	2,861 (92.4)	1,170 (92.2)
	No	235 (7.6)	99 (7.8)
Wearing shoes when go defecating/to toilets	Yes	2,748 (88.8)	1,130 (89.0)
	No	348 (11.2)	139 (11.0)
Washing hands after defecating	Yes	2,830 (91.4)	1,160 (91.4)
	No or don't know	266 (8.6)	109 (8.6)
Washing hands before eating	Yes	2,907 (93.9)	1,195 (94.2)
	No or don't know	189 (6.1)	74 (5.8)
Use of soap or ashes when washing hands	No	1,658 (53.6)	703 (55.4)
	Yes	1,438 (46.4)	566 (44.6)
Do you know anything about worms?	No	1,112 (35.9)	448 (35.3)
	Yes	1,984 (64.1)	821 (64.7)
Sources of infection with worms, number of correct answers	0	1269 (41.0)	502 (39.5)
	< 3	349 (11.3)	139 (11.0)
	3-5	868 (28.0)	357 (28.1)
	6-8	610 (19.7)	271 (21.4)
Own dog	Yes	2,461 (79.5)	1,001 (78.9)
	No	635 (20.5)	268 (21.1)
Own farm animals	Yes	2,992 (96.6)	1,240 (97.7)
	No	104 (3.4)	29 (2.3)

IQR: interquartile range;
The baseline sample (left column) includes both participants and non-participants in the cohort; the cohort sample includes all cohort participants regardless of whether they were present at one or two follow-up survey(s).
[a] For age, in case of inconsistencies in the age across years, if two of the three values were consistent then the third one was corrected so as to achieve consistency across all three values.
[b] For education attainment, the same procedure as for age was used in case of inconsistencies reported by individuals over 20 years old and not attending school anymore.
Data were obtained from a two-year cohort survey carried out among 3,096 participants at baseline (2012) and 1,269 participants at follow-up (2013 & 2014), in eight villages of Preah Vihear province, Cambodia.
