# Supplementary material for: Ivermectin Treatment and Sanitation Effectively Reduce Strongyloides stercoralis Infection Risk in Rural Communities in Cambodia
Source: PLoS Negl Trop Dis. 2016 Aug 22;10(8):e0004909. doi: 10.1371/journal.pntd.0004909 (PMC4993485; doi:10.1371/journal.pntd.0004909)
Supplement: S2 Table — OR: odds ratio; CI: confidence interval; LRT: likelihood ratio test. a: For infection risk at follow-up, the values are that of the previous year, i.e. 2012 for the first follow-up and 2013 for the second follow-up. b: For infection risk at follow-up, the baseline values of socioeconomic level were used. Data were obtained from a two-year cohort survey carried out among 3,096 participants at baseline (2012) and 1,269 participants at follow-up (2013 & 2014), in eight villages of Preah Vihear province, Cambodia. (PDF) [file pntd.0004909.s003.pdf]

S2 Table. Bivariate associations between explanatory variables submitted for variable selection and *S. stercoralis* infection risk at baseline and at follow-up

		Prevalence at baseline			Incidence at follow-up		
**Variable**	**Category**	**OR**	**95%CI**	**LRT p-value**	**OR**	**95%CI**	**LRT p-value**
Level of education [(a)]	Primary	1.00		<0.0001	1.00		0.028
	No schooling	0.72	0.57-0.91		0.55	0.33-0.93	
Occupation [(a)]	School, at home, other	1.00		<0.0001	1.00		0.099
	Rice farmer	1.53	1.32-1.78		1.25	0.96-1.62	
	Secondary or higher	1.27	1.08-1.50		1.11	0.83-1.48	
Socioeconomic level [(b)]	Least poor	1.00		0.103	1.00		0.220
	Poor	1.08	0.90-1.29		0.91	0.66-1.25	
	Poorest	1.22	1.02-1.47		1.2	0.88-1.63	
Reported regular place of defecation	Toilet	1.00		0.006	1.00		0.047
	Forest	1.15	0.95-1.40		1.20	0.86-1.67	
	Rice field or water	0.98	0.81-1.17		1.61	1.15-2.24	
	Behind the house	0.67	0.49-0.93		1.07	0.60-1.93	
Availability of toilets at home	No	1.00		0.256	1.00		0.094
	Yes	1.09	0.94-1.27		0.78	0.59-1.04	
Wearing shoes, frequency	Often	1.00		< 0.001	1.00		0.596
	Always	1.18	1.01-1.39		0.78	0.47-1.28	
	Sometimes or never	0.72	0.56-0.92		0.99	0.57-1.71	
Wearing shoes at work or school	Yes	1.00		0.009	1.00		0.876
	No	0.66	0.49-0.91		1.03	0.68-1.56	
Wearing shoes at home	Yes	1.00			1.00		0.876
	No	0.9	0.68-1.21	0.491	1.03	0.68-1.56	
Wearing shoes to go defecating/to toilets	Yes	1.00			1.00		0.035
	No	0.71	0.55-0.91	0.007	2.07	1.10-3.91	
Wearing shoes at home and/or to toilets	Any other case	n.a.	n.a.	n.a.	1.00		0.102
	No at home, yes to toilets	n.a.	n.a.	n.a.	1.81	0.59-5.49	
	No at home, not to toilets	n.a.	n.a.	n.a.	2.22	1.04-4.76	
Wearing shoes at work/school and/or to toilets	Any other case	1.00		0.014	n.a.	n.a.	n.a.
	No at work/school, yes to toilets	0.91	0.52-1.60		n.a.	n.a.	n.a.
	No at work/school, not to toilets	0.59	0.41-0.85		n.a.	n.a.	n.a.
Washing hands after defecating	Yes	1.00		0.500	1.00		0.040
	No	1.10	0.84-1.43		1.54	1.04-2.28	
Washing hands before eating	Yes	1.00		0.920	1.00		0.910
	No	1.02	0.74-1.39		0.97	0.54-1.72	
Use of soap or ashes when washing hands	Yes	1.00		0.600	1.00		0.639
	No	1.04	0.90-1.21		1.07	0.81-1.41	
Do you know anything about worms?	No	1.00		0.017	1.00		0.933
	Yes	1.22	1.04-1.43		1.02	0.70-1.47	
Sources of infection with worms, number of correct answers	0	1.00		0.030	1.00		0.569
	< 3	1.23	0.95-1.58		1.00	0.60-1.67	
	3-5	1.22	1.02-1.47		1.22	0.84-1.79	
	6-8	1.33	1.08-1.63		1.02	0.68-1.53	
Own a dog	No	1.00		0.600	1.00		0.269
	Yes	0.95	0.79-1.14		1.20	0.86-1.67	
Own farm animals	Yes	1.00		0.340	1.00		0.988
	No	0.81	0.53-1.25		0.99	0.39-2.56	
Infected at baseline	No	n.a.	n.a.	n.a.	1.00		0.124
	Yes	n.a.	n.a.	n.a.	1.25	0.94-1.67	
Number of family members at baseline	-	1.00	0.96-1.04	0.940	1.03	0.97-1.10	0.372
Proportion of houses with latrines in the village at baseline (%)		1.00	1.00-1.01	0.270	0.99	0.980-0.996	0.045
Village prevalence at baseline	-	n.a.	n.a.	n.a.	0.95	0.88-1.03	0.269

OR: odds ratio; CI: confidence interval; LRT: likelihood ratio test;
[a]: For infection risk at follow-up, the values are that of the previous year, i.e. 2012 for the first follow-up and 2013 for the second follow-up.
[b] For infection risk at follow-up, the baseline values of socioeconomic level were used.
Data were obtained from a two-year cohort survey carried out among 3,096 participants at baseline (2012) and 1,269 participants at follow-up (2013 & 2014), in eight villages of Preah Vihear province, Cambodia.
