# Supplementary material for: Ivermectin Treatment and Sanitation Effectively Reduce Strongyloides stercoralis Infection Risk in Rural Communities in Cambodia
Source: PLoS Negl Trop Dis. 2016 Aug 22;10(8):e0004909. doi: 10.1371/journal.pntd.0004909 (PMC4993485; doi:10.1371/journal.pntd.0004909)
Supplement: S3 Table — IRR: incidence rate ratio; CI: confidence interval; IRR in bold are significant at 5% level. As Poisson regression provides biased standard errors when being applied to binary data, confidence intervals were computed using Miettinen's formula IRR^(1 +/- 1.96/t) where t is the valid t-value of the respective parameter estimate in the corresponding mixed logistic regression model. (PDF) [file pntd.0004909.s004.pdf]

S3 Table. Incidence rate ratios for risk factors of *S. stercoralis* infection risk at follow-up.

Variable	Category	IRR	95% CI	p-value
Sex	Male	1.00	-	
	Female	0.82	0.65-1.04	0.120
Age (years)	Linear term	0.99	0.98-1.00	0.115
Level of education	Primary	1.00	-	
	No schooling	**0.52**	0.31-0.86	0.016
	Secondary or higher	1.13	0.88-1.45	0.372
Occupation	School, at home, other	1.00	-	
	Rice farmer	**1.50**	1.09-2.06	0.021
Reported regular place of defecation	Toilet	1.00	-	
	Forest	1.10	0.80-1.50	0.569
	Rice field or water	**1.43**	1.07-1.91	0.024
	Behind the house	1.10	0.62-1.96	0.741
Wearing shoes at home and/or to toilets	Any other case	1.00	-	
	No at home, yes to toilets	1.88	0.73-4.80	0.217
	No at home, no to toilets	**2.59**	1.30-5.17	0.010
Washing hands after defecating	Yes	1.00	-	
	No	1.33	0.95-1.87	0.126
Positive for *S. stercoralis* at baseline	No	1.00	-	
	Yes	1.14	0.89-1.48	0.338
		1.00	-	
Proportion of houses with latrines in the village (%)		**0.99**	0.980-0.997	0.006

IRR: incidence rate ratio; CI: confidence interval; IRR in bold are significant at 5% level.
As Poisson regression provides biased standard errors when being applied to binary data, confidence intervals were computed using Miettinen's formula IRR^(1 +/- 1.96/t) where t is the valid t-value of the respective parameter estimate in the corresponding mixed logistic regression model.
