# Supplementary material for: Ivermectin Treatment and Sanitation Effectively Reduce Strongyloides stercoralis Infection Risk in Rural Communities in Cambodia
Source: PLoS Negl Trop Dis. 2016 Aug 22;10(8):e0004909. doi: 10.1371/journal.pntd.0004909 (PMC4993485; doi:10.1371/journal.pntd.0004909)
Supplement: S4 Table — OR: odds ratio; CI: confidence interval; OR in bold are significant at 5% level. Data were obtained from a two-year cohort survey carried out among 1,128 participants at follow-up (2013 & 2014), in seven villages of Preah Vihear province, Cambodia. (PDF) [file pntd.0004909.s005.pdf]

S4 Table. Results of the multivariate model for *S. stercoralis* infection risk at follow-up, excluding the village with 4% sanitation coverage.

Variable	Category	OR	95% CI	p-value
Sex	Male			
	Female	0.79	0.59-1.05	0.100
Age (years)	Linear term	0.99	0.98-1.01	0.280
Level of education	Primary	1.00		
	No schooling	**0.45**	0.23-0.85	0.015
	Secondary or higher	1.10	0.81-1.50	0.549
Occupation	School at home, other	1.00		
	Rice farmer	**1.51**	1.02-2.24	0.038
Reported usual place of defecation	Toilet	1.00		
	Forest	1.05	0.74-1.50	0.782
	Rice field or water	**1.51**	1.05-2.16	0.025
	Behind the house	1.00	0.48-2.06	0.995
Wearing shoes at home and/or to toilets	Any other case	1.00		
	No at home, yes to toilets	2.58	0.82-8.13	0.107
	No at home, not to toilets	**2.61**	0.98-6.94	0.054
Washing hands after defecating	Yes	1.00		
	No	**1.58**	1.01-2.47	0.044
Positive for *S. stercoralis* at baseline	No	1.00		
	Yes	1.13	0.82-1.56	0.457
Proportion of houses with latrines in the village (%)		**0.990**	0.980-0.999	0.033

OR: odds ratio; CI: confidence interval; OR in bold are significant at 5% level.
Data were obtained from a two-year cohort survey carried out among 1,128 participants at follow-up (2013 & 2014), in seven villages of Preah Vihear province, Cambodia.
